# Practice makes perfect! Patient safety starts in medical school: Do instructional videos improve clinical skills and hygiene procedures in undergraduate medical students?

**DOI:** 10.3205/zma001224

**Published:** 2019-03-15

**Authors:** Andjela Bäwert, Anita Holzinger

**Affiliations:** 1 Medical University of Vienna, Teaching Center, Assessment and Skills, Vienna, Austria; 2 Medical University of Vienna, Teaching Center, Research Unit for Curriculum-Development, Vienna, Austria

**Keywords:** Patient safety, medical students, teaching videos, medical skills

## Abstract

**Introduction: **In 2012 safety strategies were defined in five intervention areas to improve patient safety in Austria. Regarding policy development, patient safety should be mandatory part of education of all healthcare sectors, and measures to improve hygiene standards are to be included in organizational development. The aim of this project was to achieve sustained improvement in routine procedures and anchor patient safety in the undergraduate medical curriculum by making online instructional videos on clinical skills and hygiene procedures permanently available as preparation for the first clinical clerkship.

**Method: **Short films explaining how to insert urinary catheters in women and men were produced and provided online. These videos were shown to medical students shortly before the practical Objective Structured Clinical Examination (OSCE). After viewing the videos, all of the students were surveyed using an online questionnaire with 15 questions regarding quality and acceptance. The effect of the videos on learning success was determined by the assessment outcome through red cards in the practical exam. A red card for behavior endangering the doctor or others meant zero points and discontinuation of the assessment at that particular OSCE station.

**Results: **A total of 647 students viewed one of the two videos on urinary catheters, 623 responded to the online Moodle questionnaire completly. 551 (85.2%) reported being better able to recall individual steps and procedures, 626 students (96.7%) positively rated the fact that instructional videos were available on the Medical University of Vienna’s website. More than half of the respondents (56.6%) were better able to remember critical hygiene practices. The comparison of the assessment outcomes on the OSCE for 2016 and 2013, a year in which the instructional videos were not yet available, shows no significant (chi^2^=3.79; p>0.05) but a trend towards improvement. The chance of getting a red card in 2013 was 3.36 times higher than in 2016.

**Conclusion: **Even if our study was unable to show significant improvements in the OSCE as a result of viewing the videos, it appears that clearly imparting medical skills and hygiene standards—including in visual form—is still important prior to the first clerkship to ensure the highest level of patient safety possible. The combination of teaching and learning formats, such as videos on online platforms with textbooks or lecture notes, is well suited to increase effectiveness and efficiency in learning. There is a need for further studies to investigate and analyze the effects of instructional videos in more detail.

## Zusammenfassung

**Einleitung: **Um im Bereich Patientensicherheit Verbesserungen zu erzielen wurden 2012 in Österreich Sicherheitsstrategien in 5 Interventionsfeldern festgeschrieben. Im Interventionsfeld Politikentwicklung soll die Patientensicherheit in die Ausbildung aller Gesundheitsberufe miteinbezogen werden, in der Organisationsentwicklung sollen Maßnahmen zur Verbesserung von Hygienestandards gesetzt werden. Das Bestreben dieses Projektes war es durch Darstellung klinischer und hygienischer Fertigkeiten in permanent online abrufbaren Lehrvideos, als Vorbereitung auf die erste klinische Famulatur, Handlungsabläufe nachhaltig zu verbessern und damit Patientensicherheit bereits in der medizinisch-studentischen Lehre zu verankern.

**Methode: **Kurzfilme mit der Anleitung zum Legen eines Harnkatheters bei der Frau und beim Mann wurden hergestellt und online gestellt. Diese wurden den StudentInnen kurz vor der praktischen OSCE Prüfung vorgeführt. Im Anschluss daran wurden alle StudentInnen mittels eines Online-Fragebogens, der 15 Fragen zur Qualität und Akzeptanz beinhaltete, befragt. Die Auswirkungen der Videos auf den Lernerfolg wurden durch den Prüfungsoutcome beim abschließenden OSCE („Objective Structured Clinical Examination“), indem die Anzahl der vergebenen „Roten Karten“ erhoben wurde, ermittelt. Eine rote Karte bedeutete 0 Punkte und Prüfungsabbruch bei dieser Prüfungsstation bei selbst- oder fremdgefährdendem Verhalten.

**Ergebnisse:** Insgesamt sahen 647 Studierende eines der beiden Harnkatheter-Videos, 623 beantworteten online den auf Moodle gestellten Fragebogen vollständig. 551 Studierende (85,2%) gaben an, sich besser an einzelne Handgriffe und Handlungsabläufe erinnern zu können, 626 Studierende (96,7%) bewerteten es positiv, dass Lehrvideos im Online-Tool der MedUni Wien zur Verfügung gestellt werden. Mehr als die Hälfte der Befragten (56,6%), konnten sich besser an notwendige hygienische Handgriffe erinnern. Der Vergleich des Prüfungsoutcome bei der OSCE-Prüfung des Jahrganges 2016 mit dem 2013, bei dem Instruktionsvideos noch nicht zur Verfügung standen, zeigte keine signifikante (Chi^2^=3,79; p-Wert>0,05), wohl aber eine tendenzielle Verbesserung der Studierenden. Die Chance eine Rote Karte zu bekommen war 2013 3,36 mal so hoch als im Vergleichsjahr 2016.

**Fazit: **Wenngleich in unserer Studie keine signifikanten Ergebnisse durch das Vorführen der Videos in der OSCE Prüfung selbst zu verzeichnen waren, so erscheint uns die regelrechte – auch visuelle – Vermittlung von ärztlichen Fertigkeiten und hygienischen Standards vor der ersten Famulatur wichtig um größtmögliche Patientensicherheit zu gewährleisten. Die Kombination aus Lehr- und Lernformaten, wie Videos auf Online-Plattformen, in Verbindung mit Lehrbüchern oder Skripten, erscheint uns gut geeignet um Effektivität und Effizienz beim Lernen zu erhöhen. Es bedarf weiterer Studien um die Effekte von Lehrfilmen besser belegen zu können.

## Introduction

Patient safety encompasses by definition the measures taken to prevent adverse events which could harm the patient and has been identified as the top healthcare priority in European healthcare policy [1]. Although there are many safety requirements laid down by law, including legislation governing medicinal products and devices, protections against communicable disease, and mandatory quality assurance, there is a lack of institution-wide risk and error management systems and their monitoring, as well as no appropriate strategy for their implementation.

For this reason in 2012 a national strategy for patient safety was developed in Austria following the capacity building model [2]. According to this model, there are five areas in which measures affecting patient safety can be implemented and goals defined: organizational development, personnel development, monitoring, raising public awareness and policy development. In policy development, the area defining measures for decision-makers, one of the measures to be implemented is the inclusion of patient safety in the undergraduate and post-graduate education of all regulated healthcare occupations. Within organizational development, the area defining the establishment of clinical risk management as the most important strategic measure, “recognized systems” to document nosocomial infections are to be implemented as part of developing more user-friendly information and communication technologies. Within the scope of implementing safe practices to prevent the most commonly occurring incidences, e.g. therapy-related infections, special importance is to be placed on hand hygiene and the avoidance of antibiotic resistance [3].

Following these two specific areas of intervention, our study focuses on how patient safety can be anchored in undergraduate medical education. Connected to this are both the teaching of hygiene standards in basic medical training and the imparting of practical clinical skills, all content found in the skills line “Basic Medical Skills” (*Ärztliche Grundfertigkeiten*) taught in the second year of study at the Medical University of Vienna. Since there can be long intervals of time between learning skills and performing them on patients, there needs to be more focused measures to ensure a sustained ability to remember specific skills. The opportunity to repeat a skill, such as proper hand disinfection before inserting a catheter or the separate steps to insert the catheter, has been limited to re-reading the practical training notes. However, numerous studies show that two-dimensional demonstrations of medical procedures alone are not sufficient to enable students to remember manual steps exactly. Instructional videos with three-dimensional presentation of information would be a valuable supplementary teaching aid when a precise sequence of steps needs to be taught [4].

This study aims to improve confidence in performing procedures and reducing the frequency of errors by using instructional videos that give precise instructions on how to properly disinfect hands and insert a urinary catheter in men and women.

The study questions asked were:

1. Was the assessment outcome better in the student cohort that viewed the instructional videos in comparison with a student cohort that only had access to written material?
2. Do students remember individual steps better?
3. Do students understand hygiene procedures better?
4. What is the acceptance of these videos?

## Method

In parallel to the existing written course materials, videos were produced for the skills line “Basic Medical Skills”:

1. Donning and removing sterile gloves
2. Hygienic disinfection of hands
3. Hand disinfection prior to performing surgery
4. Urinary catheter insertion in men
5. Urinary catheter insertion in women

Core components of the course include proper technique and hygienic behavior to optimally protect the patient and prevent any danger to the physician or others within the scope of the medical procedure. The main goal is optimal preparation of students for their first required clerkship that is served in a hospital, outpatient clinic or doctor’s office. Each year 660 medical students and 80 dental students are taught in small groups of 10-12.

The selection of the topics that were filmed was based on the test results of the previous years. The medical skills chosen were ones that students had tended to earn fewer points at the practical exam according to errors in hygiene procedures that posed a possible danger to patients, along with deficiencies in the performance.

In our study, the two videos on how to insert an urinary catheter women and men were shown to students for the first time in June 2016. To ensure that all study participants were subject to the same conditions, the videos were made available to 647 study participants for one-time viewing in the computer learning studio (CLS) at the Medical University of Vienna. Two parallel groups viewed the two instructional videos back-to-back in two computer learning studios via their own Moodle access at the beginning of the three-week-long practice period prior to the final OSCE.

After viewing the instructional videos, students were asked to fill out an online questionnaire on Moodle with 15 questions. Questions about video quality and acceptance were asked (see table 1 (Tab. 1)).

The assessment outcome was determined by the number of red cards given for behavior endangering the physician or others and resulted in a score of 0 points for the particular OSCE station. This number was compared with the assessment results for previous years. As part of the relevant training, events leading to a red card were defined in advance by the course planning team and explained to the instructors who also serve as OSCE assessors.

On average there is a period of eight months between learning of the basic medical skills and the OSCE. Students complained that after completion of the courses, the skills demonstrated and practiced could only be referred to or read about in the written scripts. A repeat demonstration or practice session was only possible during the three-week preparatory phase immediately prior to the OSCE. The critical aspect for students was that, although the official learning material contained detailed instructions and diagrams for each procedure, they did not reflect fully fluid sets of movements because they were two-dimensional and thus not sufficient for long-term memory of the medical skills or hygienic practices. To address this problem, it appeared to us that the combination of electronic teaching material with the written learning scripts was well suited to increase effectiveness and cognitive retention. Thus students should be enabled to recall medical skills with less complication at a later time during their studies, for instance, prior to the practical clinical year.

In addition, data on video quality and acceptance was gathered via an online student survey. This information is meant to serve as a basis to design future instructional videos more efficiently and broaden their use. How these videos affect students’ skills was measured by the assessment outcome, which was then compared with the outcomes of previous years.

## Results

Overall, 647 students viewed one of the two videos on urinary catheters, 318 (49.1%) watched the video on men and 329 (50.9%) the video on women. A total of 623 students (96.3%), of which 317 women (50.9%) and 306 men (49.1%), responded to the questionnaire in full. The mean age for men was 21.67 years, and for women 21.3 years. Twenty-four respondents (3.7%) either did not fill out the questionnaire completely or did not respond.

The questionnaire contained 15 questions on a three-point scale (“I don’t know,” “I agree,” “I disagree”). The videos received a high level of approval in terms of imparting practical skills. The qualitative analysis showed that offering the videos as supplementary learning tools was rated positively. Almost all of the study participants reported being better able to recall the individual steps after watching the video, and more than half could better remember the hygienic techniques. Almost all of the students were in favor of the videos as supplementary material and desired more instructional videos (see table 1 (Tab. 1): Questionnaire).

The assessment outcome was measured by the number of red cards. In 2013 the test asked 114 students to insert an urinary catheter. Of these, 14 students (12.3%) received a red card. In contrast, only three of 75 possible red cards (4.0 %) were given in 2016 for errors involving urinary catheter insertion. The year of study had no significant influence on the giving of red cards (chi-square=3.79; p>0.05). The odds of receiving a red card in 2013, however, were 3.36 times higher than in 2016 (see table 2 (Tab. 2)).

## Discussion

The aim of this study was to establish measures in medical education to ensure patient safety [5]. Teaching materials for the “Basic Medical Skills” course have existed for many years in the form of written texts. Our study supplemented these teaching material with three-dimensional presentations. With the specially produced videos, we intended to demonstrate the best techniques to disinfect hands and the practical steps to insert urinary catheters. The goal was to offer students the opportunity to individually review these actions in the proper sequences to make recalling the learned steps easier.

In our study a slight tendency was seen among students who had the possibility to additionally watch the videos for repetition to score better on the OSCE in comparison to students in previous years without access to the instructional videos. International studies focused on the demonstration of practical clinical skills often show clearer findings. For example, in surgery such videos have been long in use. Davidson et al. analyzed the data of a website on which topics and videos from neurosurgery were made available to improve surgical techniques in neurosurgery and increase patient safety. It was demonstrated that from 2016 to 2017, there were 246,259 website hits and videos were downloaded 143,868 times. Most frequently, 25- to 34-year-olds took advantage of the opportunity to learn additional material about neurosurgery online. These research findings point out that freely accessible online portals are well accepted as a learning tool and that there is a need for additional online resources for continuing education [6]. Similar to our study, Pilieci et al. investigated whether showing of videos in which medical skills such as hand disinfection prior to surgery, donning sterile gloves or hand disinfection were shown with precision, led to an improvement in the clinical skills of first-semester medical students. The aim of this study was to gather data on student acceptance of visual teaching formats and to search for a possibility to better teach medical skills and hygiene standards and, in turn, to increase patient safety by preventing post-operative infections. The students in this study favored videos if it involved easy access to the teaching tool, simplicity of use, and the unrestricted option to repeat the individual steps. According to the results of this study, instructional videos featuring medical skills and hygiene practices can provide a significant contribution to reducing post-surgical infections and increasing patient safety [7].

Since a great challenge in practicing medicine is posed by multiresistant pathogens caused by antibiotic resistance, learning hygienic practices and techniques is critical, especially when inserting catheters. As shown by many studies, proper hand hygiene before touching the patient lowers the risk of infection by up to 30% [8], [9], [10].

In the GMA catalogue of learning objectives, hygiene is listed as a learning objective under 1f level 3 and defined as a student being familiar with and able to identify common hygiene errors and their effect on patient safety. In addition, the student is able to reflect on hygiene practices as they pertain to routine medical practice. The student knows that specific clinical standards for hygiene exist [11]. Our study confirms that the combination of e-learning formats, such as online videos, with conventional formats, such as textbooks or lecture notes, is well suited to increase effectiveness and sustainability when learning practical skills and proper hygiene practices. This is a simple, cost-effective method to demonstrate previously learned material in a manner true to detail. The unlimited opportunity to view the videos repeatedly contributes to patient safety early on during medical study.

Already at the end of the 1990s, healthcare policy began to turn more attention to error management and transparency in the healthcare system [12]. While patient safety measures, such as quality monitoring, definition of hygiene standards, studies on drug interactions and much more, began to show concrete results in clinical practice, anchoring the topic of patient safety into the medical curriculum and the curricula of the other healthcare professions lagged behind. In 2010 an expert group from the WHO’s World Alliance of Patient Safety drafted a patient safety curriculum guide for medical schools [13]. Since then and even before, measures have been taken at various German-speaking universities to integrate this topic into teaching. A concrete example is found in the Catalogue of Learning Objectives regarding Patient Safety in Undergraduate Medical Education *(Lernzielkatalog Patientensicherheit für das Medizinstudium;* GMA-LZK), developed by the GMA committee on patient safety and error management, and which has found timely inclusion in the National Catalogue of Competency-based Learning Objectives in Undergraduate Medical Education (NKLM) [http://www.nklm.de].

In Austria, the Federal Ministry of Health developed a strategy for patient safety to minimize adverse events. A platform for patient safety was created to address current topics and projects dealing with patient safety; a post-graduate course of study in patient safety and quality in the healthcare professions exists at the Medical University of Vienna [https://www.plattformpatientensicherheit.at/]. Recently, initiatives have been called into being to encourage and promote scientific collaboration on patient safety. For instance, there are regular journal clubs at the Medical University of Vienna in which our instructional video project and other projects on interprofessionalism in pediatric simulation, medical simulation and emergency management or simulation training sessions on vacuum-assisted delivery have been presented with the aim of establishing patient safety as a topic in teaching and research.

According to our research, instructional videos abound online; studies investigating the direct effect of instructional videos on clinical skills and hygiene practices or the effect on learning success are rare, however. Randomized, case-control studies are needed to gather supporting evidence for this method of imparting knowledge.

Among the limitations of this study is the absence of a control group for 2016. We only compared different years. A detailed investigation of assessment outcomes would be valuable to show a differentiated improvement in skills. Our study only included the complete failure of students at the OSCE stations in its analysis.

## Competing interests

The authors declare that they have no competing interests. 

## Figures and Tables

**Table 1:**
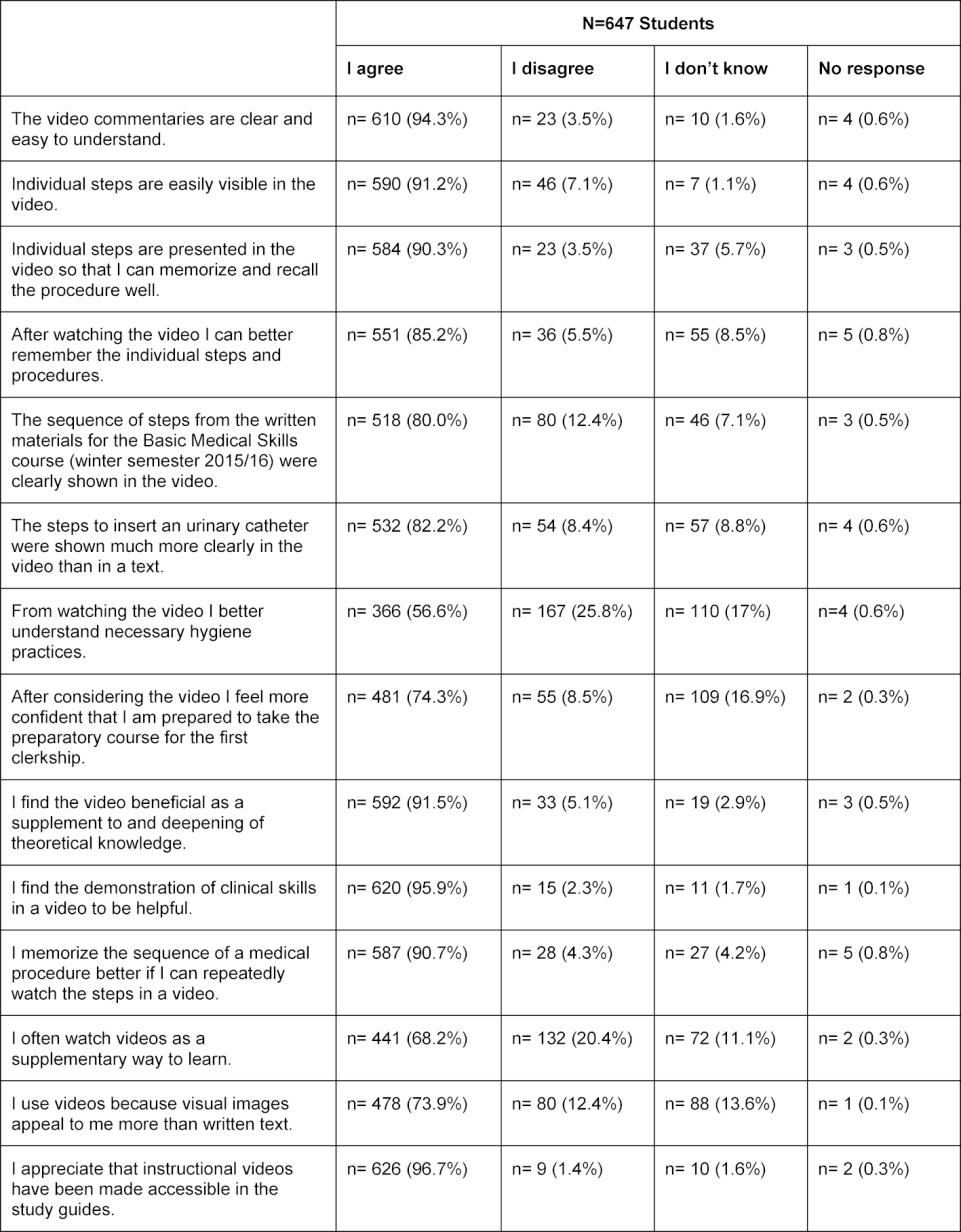
Questionnaire

**Table 2:**
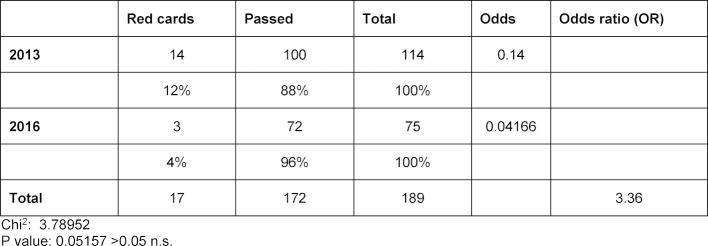
Assessment outcome: Red cards in 2013 versus 2016
